# SARS-CoV-2-specific T-cells in unexposed humans: presence of cross-reactive memory cells does not equal protective immunity

**DOI:** 10.1038/s41392-020-00338-w

**Published:** 2020-10-06

**Authors:** Rory D. de Vries

**Affiliations:** Department Viroscience, Erasmus MC, Rotterdam, the Netherlands

**Keywords:** Infectious diseases, Respiratory tract diseases

Using human blood samples obtained from pre-pandemic donors, a recent article by Mateus et al. in *Science* provided new evidence that SARS-CoV-2-reactive T-cells in unexposed donors are indeed HCoV-specific T-cells.^1^

The rapid global spread of coronavirus disease 2019 (COVID-19), caused by the newly-emerged coronavirus SARS-CoV-2, has led to millions of infections with substantial morbidity and mortality.^2^ Different clinical manifestations of COVID-19 have been observed: asymptomatic infections, mild self-limiting disease, acute respiratory distress syndrome and death. The determinants underlying disease severity currently remain elusive; since severe patients often present with immune hyperresponsiveness, it is speculated that the host’ immune response could be a contributing factor to severe disease.

Many studies are dissecting the human immune response to SARS-CoV-2 and several groups have reported marked activation of T-cell subsets in acute COVID-19 patients.^3^ The antigen-specific T-cell response has only been analyzed in a handful of papers, all sharing a common feature: although SARS-CoV-2-specific CD4^+^ and CD8^+^ T-cells are consistently detected in peripheral blood mononuclear cells (PBMC) obtained from COVID-19 patients, studies also report activation of T-cells in 20–50% of the people never exposed to SARS-CoV-2.^1,4,5^ The frequency of these cross-reactive responses in pre-pandemic controls is always low. On average 1% of the CD4^+^ T-cells from acute COVID-19 patients upregulate activation markers upon peptide pool stimulation. If pre-pandemic donors respond to peptide stimulation, the percentage of responding CD4^+^ T-cells is always <0.1%.^5^ Authors of these papers speculate that these–mainly CD4^+^–SARS-CoV-2-reactive T-cells are probably induced by past infection with one of the endemic “common cold” coronaviruses (HCoVs), which share at least partial sequence homology with SARS-CoV-2. Experimental evidence for this hypothesis was lacking so far.

In this recent article in *Science*, the authors provided new evidence that SARS-CoV-2-reactive T-cells in unexposed donors are indeed HCoV-specific T-cells.^1^ The authors relied on an elegant design of peptide “megapools” (MPs) to determine T-cell specificities. Using these peptide pools in combination with human PBMC obtained from donors between 2015 and 2018, the authors identified 142 HLA class II-restricted SARS-CoV-2 T-cell epitopes. Generation of several short-term T-cell lines proved that at least a number of these T-cells recognized peptides derived from HCoVs.

This research group previously developed the MP approach to characterize T-cell responses targeting many different viruses in vaccine recipients and convalescent donors (including SARS-CoV-2), allowing them to simultaneously test large numbers of epitopes even when sample size is limited.^4,5^ In this study, the authors initially expanded PBMC from pre-pandemic donors by stimulation with two different MPs: MP_CD4_S (containing predicted epitopes of the S protein) and MP_CD4_R (containing predicted epitopes of all other proteins). After expansion, T-cell reactivity to smaller pools was determined, followed by reactivity to individual peptides, a process known as deconvolution. This led to the identification of 142 novel SARS-CoV-2 epitopes. Most frequent and most vigorous responses were observed with antigens from the S protein, although the mapped epitopes were distributed fairly even over the proteome in proportion to protein size. In addition to S, responses to ORF6, ORF3A, N, ORF8 and within ORF1a (nsp3, nsp12, nsp4, nsp6, nsp2, and nsp14) were prominently present. Antigen-specific cells responding to peptide pool stimulation were mainly CD4^+^ T-cells. This was not surprising, as the MPs employed contained long peptides (15 amino acids) of predicted class II epitopes.

Although the experimental approach employed by the authors (2-week expansion after direct peptide stimulation) allows for *de novo* generation of responses from naive T-cells, they recognized that the reactivity observed might reflect the presence of memory T-cells cross-reactive between HCoVs and SARS-CoV-2. To study this, the authors split the peptides used in these studies into 3 groups: (1) not immunogenic, (2) immunogenic in 1 donor, and (3) immunogenic in multiple donors. In this analysis, they found that peptides immunogenic in multiple donors indeed shared a higher degree of amino acid homology between circulating HCoVs and SARS-CoV-2.

Focusing on cross-reactive responses the authors now generated new MPs. For these cross-reactive MPs they selected all epitopes with >67% amino acid homology and the highest responders. In this way, they made a MP containing 31 “cross-reactive” S epitopes (MP_CD4_S31) and a MP containing 30 “cross-reactive” epitopes spanning the rest of the proteome (MP_CD4_R30). Simultaneously, they generated two other MPs containing the homologous epitopes derived from different HCoVs. They named these the MP_CD4_S124 and MP_CD4_R129 (containing 124 and 129 epitopes, respectively). These four MPs were tested in two different cohorts, unexposed donors and COVID-19 convalescent donors. In these experiments, both cohorts respond equally well to the HCoV MPs. The unexposed cohort still responded to the SARS-CoV-2 pool, but not in the magnitude observed in COVID-19 convalescent donors. These data are consistent with the hypothesis that cross-reactive CD4^+^ T-cells between SARS-CoV-2 and other HCoVs exist in many individuals.

For the final experiments, the authors generated 42 short-term T-cell lines by stimulating PBMC with epitopes from the cross-reactive SARS-CoV-2 MPs (S31 and R30). After generation of T-cell lines, these were stimulated with a dilution series of the recognized peptides obtained from either SARS-CoV-2 or the other HCoVs. Ten out of 42 T-cell lines were identified as cross-reactive. Similar experiments were performed with purified memory and naïve T-cells, to prove that cross-reactive responses in unexposed donors are derived from memory cells and were not generated de novo.

The presence of T-cell-based cross-reactive immune memory in unexposed donors had been reported by several studies, but the basis for this memory remained speculative.^4,5^ This is the first study that provides direct evidence that cross-reactive T-cell responses could be induced by infection with any of the circulating HCoVs: OC43, 229E, HKU1 or NL63.^1^

**Fig. 1:**
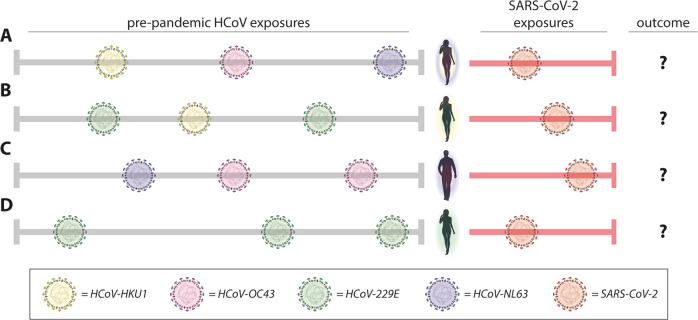
Pre-existing cross-reactive T-cell immunity could impact COVID-19 disease severity. Mateus et al. show the presence of pre-existing T-cell immunity in pre-pandemic donors (donor **a**–**d**), induced by one or multiple of the seasonal HCoVs (left part of figure). It is still unclear how pre-existing immunity impacts disease severity (or outcome) after SARS-CoV-2 exposure (right part of the figure). Different pre-exposure history could lead to different cross-reactive T-cell responses and different disease severity

Virus-specific responses of the immune system are often termed “immunity.” Although Mateus et al. clearly show the presence of cross-reactive T-cell immunity to SARS-CoV-2 in unexposed donors, it is important that this terminology is not misinterpreted as “protective immunity.” The pre-existing cross-reactive immunity (or better: immune memory) may impact COVID-19 disease heterogeneity in different ways. At the moment it is unclear whether this is a positive or negative contribution, pre-existing cross-reactive T-cells could either ameliorate or worsen COVID-19 (Fig. 1). Clinical studies to answer this question are not easy, but future prospective studies in which PBMC samples pre- and post-COVID-19 are obtained from human donors are required. The study by Mateus et al. makes clear that understanding the role of T-cells in COVID-19 disease severity is crucial to inform vaccine design and evaluation.
